# Exploring the molecular mechanisms and immune cell responses in brucellosis: Insights from gene expression profiles and immune cell scores

**DOI:** 10.1371/journal.pone.0330840

**Published:** 2025-09-25

**Authors:** Xiaoyuan Hu, Yuefei Li, Jiangshan Zhao, Yahetikezi Muhedaner, Kai Yang, Mengzhen Dong, Mukedaisi Aihemaijiang, Shuqin Lei, Jingxuan Sun

**Affiliations:** 1 Emergency Mouse Control Center, Xinjiang Uighur Autonomous Region Center for Disease Control and Prevention, Urumqi, Xinjiang, China; 2 School of public health, Xinjiang Medical University, Urumqi, Xinjiang, China; 3 Xinjiang Uighur Autonomous Region Center for Disease Control and Prevention, Urumqi, Xinjiang, China; 4 Chengdu for Disease Control and Prevention, Chengdu, Sichuan, China; Beni Suef University Faculty of Veterinary Medicine, EGYPT

## Abstract

**Background:**

Brucellosis is a typical zoonotic disease. The study aimed to identify key molecular markers and immune cell imbalances in brucellosis by integrating transcriptomic profiling, co-expression network analysis, and immune cell scoring.

**Methods:**

Gene expression profile of 103 patients with brucellosis and 46 healthy controls were obtained from the GSE69597 dataset. Differential analysis was conducted on gene expression and immune cell score, followed by co-expression network construction. Diagnostic value of module genes was assessed using Gradient Boosting Machine (GBM) and Random Forest (RF) models to screen marker genes. Peripheral blood samples from 88 brucellosis patients and 70 healthy individuals were collected for validation using flow cytometry, RT-qPCR and Western blot.

**Results:**

A total of 4924 DEGs were identified, and 11 co-expression modules were constructed. The Brown module showed the highest positive correlation with normal controls, while the Greenyellow module had the highest negative correlation. Enrichment analysis revealed that genes in the Brown module were mainly involved in the cell cycle and virus infection, genes in the Greenyellow module were primarily associated with the PI3K-Akt and Wnt signaling pathway. CDK1, MAPK11, and PDIA3 were identified as marker genes with high importance in both models. The marker genes were significantly higher expression in brucellosis. CD8 + T cells and NK cells were higher in brucellosis than in the control group, whereas B cells and CD4 + T cells were lower, which was confirmed by flow cytometry.

**Conclusion:**

The abnormal levels of CDK1, MAPK11, PDIA3, and immune cells in brucellosis may be involved in the disease’s pathogenic mechanisms.

## Introduction

Brucellosis is a global zoonotic disease caused by bacteria of the genus *Brucella* [1]. The *Brucella* genus consists of Gram-negative, non-flagellated, non-spore-forming bacteria that can survive and reproduce within a host [2]. The disease affects various domestic animals such as cattle, sheep, goats, and pigs, and can be transmitted to humans through direct contact or consumption of contaminated animal products, posing significant public health problems worldwide, especially in developing countries [3]. Brucellosis is characterized by non-specific symptoms such as fever, fatigue, night sweats, and muscle pain, and in severe cases, it can lead to complications like arthritis and endocarditis [4]. The incubation period for human brucellosis can vary from days to months, and its broad clinical presentation may overlap with other diseases, leading to misdiagnosis and delayed diagnosis [5].

The molecular mechanisms of brucellosis have not been fully elucidated. Brucella can evade host immune surveillance by inhibiting host cell apoptosis, promoting its own internalization, and hindering inflammatory responses to maintain its survival within the host [6]. Moreover, *Brucella* can affect the signaling pathways of host cells, further promoting the survival and proliferation of the bacteria [7]. Studies on the molecular and cellular mechanisms during the *Brucella* infection process not only help to clarify the complexity of the interactions between *Brucella* and its host but are also crucial for developing more effective diagnostic, preventive, and therapeutic strategies [8].

Although prior research has characterized some immunological features of brucellosis, there remains a lack of integrated analyses linking transcriptomic alterations with immune cell dysregulation and diagnostic biomarker discovery. In particular, the associations between specific gene co-expression modules and immune cell subtypes in brucellosis have not been systematically studied. Therefore, this study aimed to fill this gap by integrating gene module analysis with immune cell profiling and machine learning, and further validating key findings in clinical samples. This approach enables the identification of potential diagnostic markers and immune-related pathways relevant to *Brucella* pathogenesis.

## Materials and methods

### Data collection

GSE69597 including gene expression profiles of whole blood from 103 patients with brucellosis, and 46 healthy controls. Sample clinical information includes response to treatment (complete remission) and reinfection. The limma package was used for background correction, standardization processing, and quality control.

### Differential analysis

Differential gene expression between patients with brucellosis and healthy control groups was compared using the limma package, setting the significance level at P < 0.05. The ggplot2 package was used to create volcano plots and heatmaps, visually displaying the differentially expressed genes.

The relative abundance of immune cell subpopulations in patients with brucellosis and healthy control groups was evaluated using the CIBERSORT algorithm. The immune scores between patients with brucellosis and healthy control groups were compared, and differential analysis was conducted using the limma package to select significantly altered immune cell subpopulations. The significance level was set at P < 0.05.

### Co-expression network construction

A weighted gene co-expression network analysis (WGCNA) package was used to construct a co-expression network and identify functionally related gene modules. The pickSoftThreshold function in the WGCNA package was utilized to select the optimal soft threshold by analyzing the scale-free topology fit index for different soft thresholds, thus ensuring the network met the criterion of a scale-free network (R^2^ > 0.8). Pearson correlation coefficients were calculated for the expression similarity between all gene pairs. The similarity matrix was transformed into a weighted adjacency matrix using the selected soft threshold by calculating the connection strength between gene pairs. This weighted adjacency matrix was further transformed into a topological overlap matrix (TOM). Gene modules were identified using hierarchical clustering with the dynamic tree cut algorithm, setting a minimum module size of 30 genes. Closely related modules were merged based on a module eigengene dissimilarity threshold of 0.25. Pearson correlation analysis was used to examine the relationship between module genes and clinical traits.

The clusterProfiler package was used for GO (Gene Ontology) and KEGG (Kyoto Encyclopedia of Genes and Genomes) enrichment analysis within modules, identifying the biological processes, cellular components, molecular functions, and signaling pathways these genes are involved in, revealing their potential biological significance.

### Machine learning for participant classification

To identify marker genes with high diagnostic value, we constructed Gradient Boosting Machine (GBM) and Random Forest (RF) models using the scikit-learn and XGBoost libraries in Python. Module genes as input features, with disease status (brucellosis vs. healthy control) as the output label. To assess model robustness and predictive performance, we employed 10-fold cross-validation. The dataset was randomly partitioned into 10 subsets; in each iteration, 9 subsets were used for training and 1 for testing. This process was repeated 10 times to obtain average performance metrics. Model accuracy was evaluated using receiver operating characteristic (ROC) curves and the area under the curve (AUC). Feature importance rankings were derived from both models to identify marker genes.

### Clinical sample collection

A total of 88 patients with brucellosis and 70 healthy controls were recruited from May 20, 2023 to May 10, 2024 in the Xinjiang Uighur Autonomous Region Center for Disease Control and Prevention. To ensure the inclusion of individuals who meet the specific criteria for our research, we implemented a structured recruitment process.

Eligible participants for patients with brucellosis were identified based on typical clinical symptoms (fever, night sweats, fatigue, or joint pain) and a Standard Agglutination Test (SAT) ≥1:100. For cases with SAT titers below 1:100 but high clinical suspicion (e.g., possible early or chronic infection), blood culture for *Brucella* spp. were employed. Only individuals with either SAT ≥ 1:100 and compatible symptoms, or microbiological confirmation by culture, were included in the study. Exclusion criteria included severe cardiac, liver, kidney diseases, or other significant chronic diseases; co-infection with other infectious diseases; treatment for brucellosis or the use of medications that could affect the immune response (such as corticosteroids, immunosuppressants) within the past month; and women who were pregnant or breastfeeding. This study was approved by the Institutional Review Board and Ethics Committee of Xinjiang Uighur Autonomous Region Center for Disease Control and Prevention (2023−009) and was conducted in accordance with the Declaration of Helsinki. Informed consent was obtained from all participants prior to their involvement in the study. The consent process was designed to ensure that participants understood the study’s purpose, procedures, potential risks, and benefits. Participants were provided with a detailed consent form that explained the nature of the research, confidentiality measures, and their right to withdraw at any time without penalty.

### Flow cytometry

Peripheral blood mononuclear cells (PBMCs) were isolated from 88 brucellosis patients and 70 healthy controls using Ficoll density gradient centrifugation. For surface staining, cells were incubated for 30 minutes in the dark at 4°C with the following fluorophore-conjugated antibodies (all from BD Biosciences): CD45-PC7, CD3-PC5.5, CD4-FITC, CD8-PE, CD19-FITC, and CD56-PE. After washing, cells were resuspended in PBS containing 1% BSA and analyzed on a BD FACSCanto II cytometer. Gating strategy involved the following steps: Side scatter (SSC) to exclude debris; CD45 + population selected to define total leukocytes. Subsequent gates were set as CD3 + CD4+ for CD4 + T cells, CD3 + CD8+ for CD8 + T cells, CD3 − CD19+ for B cells, CD3 − CD56+ for NK cells. Data were analyzed using FlowJo v10 (Tree Star). The percentage of each immune cell type was calculated relative to total CD45 + cells.

### Real-time quantitative PCR (RT-qPCR)

Total RNA was extracted from blood samples of 10 brucellosis patients and 10 controls using Trizol (Invitrogen, CA, USA). The RNA concentration was measured using a NanoDrop spectrophotometer, and the integrity and purity of RNA were assessed via 1% agarose gel electrophoresis. The extracted total RNA was used as a template to synthesize cDNA using the SuperScript III First Strand Synthesis System (Invitrogen) with random primers. The cDNA, specific primers, SYBR Green qPCR reagent (Invitrogen), and the qPCR reaction mixture were combined in PCR tubes and subjected to qPCR reactions on a 7300 real-time PCR system (Applied Biosystems). The sequences of the specific primers are listed in S1 Table. The relative gene expression levels were calculated using the 2^-ΔΔCt^ method, with GAPDH selected as the internal reference gene for normalization.

### Western blot

Protein was extracted from samples of 10 brucellosis patients and 10 controls using a protein extraction kit (Beyotime, China) and the protein concentration was determined using a BCA protein assay kit (Beyotime). Equal amounts of protein were loaded onto SDS-PAGE for electrophoresis. Subsequently, the protein was transferred from the gel to a PVDF membrane in transfer buffer. The membrane was incubated with 5% non-fat milk for blocking and washed with PBS. The membrane was incubated overnight at 4°C with specific primary antibodies (Abcam, CA, UK), followed by washing with PBS. The membrane was then incubated for 1 hour at room temperature with HRP-conjugated secondary antibodies (Abcam). The signal was developed using an ECL detection system. GAPDH was used as an internal reference, and the relative expression levels of the target proteins were assessed using ImageJ software (NIH, MD, USA).

### Statistical analysis

All statistical analyses were performed using GraphPad Prism (v9.3.0, GraphPad Software, USA). Student’s t-test or one-way ANOVA was used to compare outcomes between groups. The significance level was set at P < 0.05.

## Results

### Identification of differential genes and immune cells

**Fig 1:**
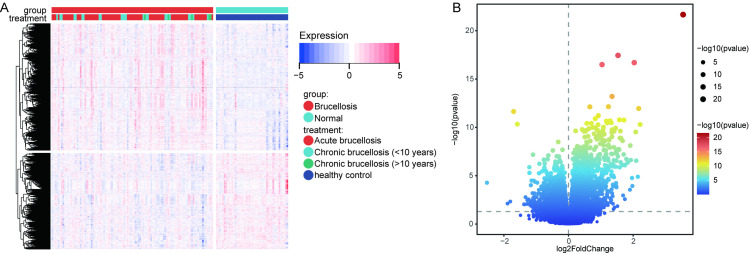
Differential analysis of genes expression in brucellosis and normal controls. **(A)** Heatmap displaying the expression of 4924 DEGs between 103 brucellosis patients and 46 controls. **(B)** Volcano plot showing 4924 DEGs, including 2510 upregulated and 2414 downregulated genes.

**Fig 2:**
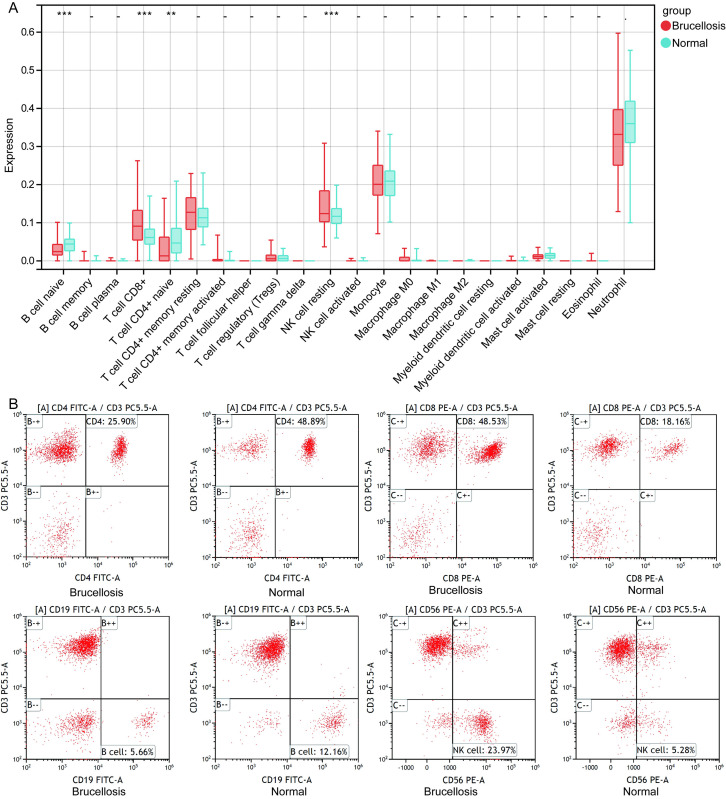
Differential analysis of immune cells in brucellosis and healthy controls. **(A)** CIBERSORT analysis showed significantly increased CD8 + T cells and NK cells, and decreased B cells and CD4 + T cells in brucellosis. **P < 0.01, ***P < 0.001. **(B)** Flow cytometry validation of increased CD8 + T cells and NK cells, and decreased CD4 + T cells and B cells in brucellosis patients. CD4 + T cells (CD3 + CD4+), CD8 + T cells (CD3 + CD8+), B cells (CD3 − CD19+), and NK cells (CD3 − CD56+) were gated from CD45 + leukocytes.

Through the analysis of the GSE69597 dataset, we identified 4924 DEGs, including 2510 upregulated and 2414 downregulated genes (Fig 1A and 1B). Based on the results from CIBERSORT, we identified a significant increase in the abundance of CD8 + T cells and NK cells in patients with brucellosis compared to the normal control group, while the abundance of B cells and CD4 + T cells was lower (Fig 2A). This result was further validated by flow cytometry (Fig 2B). Compared to the control group, the proportion of CD8 + T cells and NK cells was higher in patients with brucellosis, while the proportion of B cells and CD4 + T cells was lower.

### Construction of co-network

**Fig 3:**
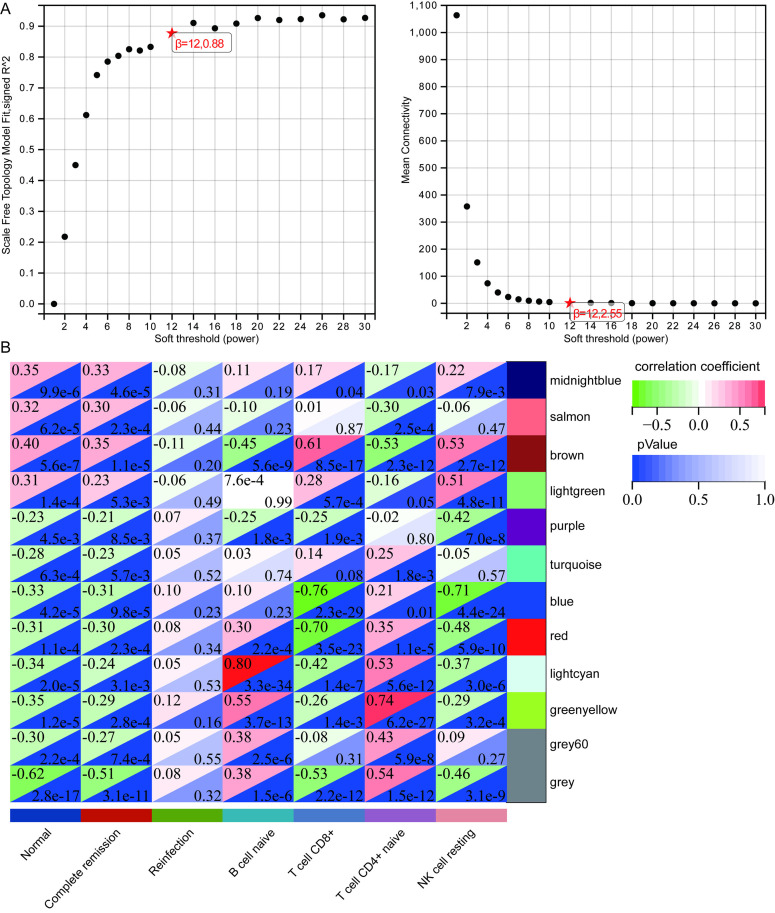
Construction of co-expression network. **(A)** The scale independence and the mean connectivity of the WGCNA analysis of DEGs. The soft-thresholding power = 12 was selected as it achieved a scale-free topology fit index R^2^ = 0.88. **(B)** Heatmap showing correlations between module eigengenes and clinical traits (disease status, remission, and immune cell types). The Brown module showed a strong positive correlation with CD8 + T cells and healthy controls, while the Greenyellow module correlated with disease state and CD4 + T cells.

In the construction of the co-expression network, selecting an appropriate soft threshold (power) was crucial to ensure the network closely matched the characteristics of a scale-free network. We set the soft threshold to 12, achieving a scale-free topology fit index of 0.88 (Fig 3A). Using WGCNA, genes with similar expression patterns were clustered into modules, resulting in 11 gene modules. Correlation analysis showed that the Brown module had the highest positive correlation with normal controls, complete remission, and CD8 + T cells, while the Greenyellow module displayed the highest negative correlation with normal controls and the highest positive correlation with CD4 + T cells (Fig 3B). This finding suggests that different gene modules may play varied roles in the progression of the disease.

### Biological functions of module genes

**Fig 4:**
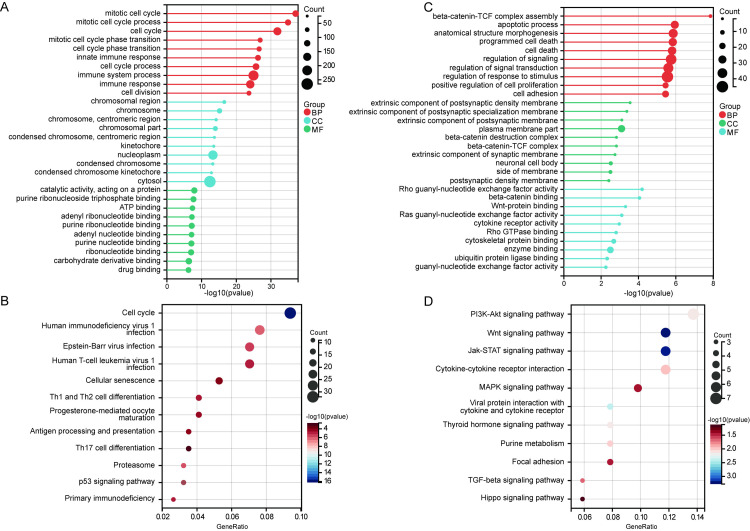
Functional enrichment analysis of key modules. **(A)** The main GO enrichment terms enriched by brown module genes, including mitotic cell cycle, and immune system process. **(B)** The main KEGG enrichment terms enriched by brown module genes, including cell cycle, and viral infection pathways. **(C)** The main GO enrichment terms enriched by greenyellow module genes, including apoptosis, and cell death. **(D)** The main KEGG enrichment terms enriched by greenyellow module genes, including Wnt, and PI3K-Akt signaling pathways.

Functional enrichment analysis of the modules revealed that genes in the Brown module were mainly involved in biological functions such as the mitotic cell cycle and immune system processes (Fig 4A). KEGG results indicated that the Brown module genes were primarily associated with the cell cycle and viral infection processes (Fig 4B). Genes in the Greenyellow module were significantly involved in biological functions like beta-catenin-TCF complex assembly and the apoptotic process (Fig 4C). In KEGG, the PI3K-Akt signaling pathway and Wnt signaling pathway were predominantly enriched by Greenyellow module genes (Fig 4D).

### Identification of diagnostic marker genes

**Fig 5:**
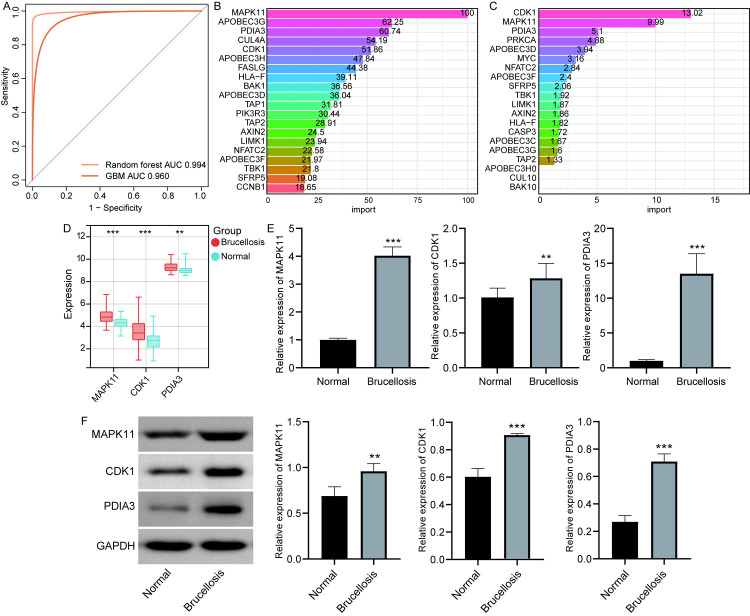
Identification and validation of diagnostic marker genes. **(A)** ROC curves showing the diagnostic performance of the GBM and Random Forest models. Based on 10-fold cross-validation, the AUC was 0.960 for GBM and 0.994 for random forest. Feature importance ranking of DEGs using GBM model (**B**) and random forest model (**C**) identified CDK1, MAPK11, and PDIA3 as top markers. **(D)** CDK1, MAPK11, and PDIA3 showed significantly higher expression in brucellosis samples from GSE69597 dataset. **(E)** RT-qPCR confirmed elevated mRNA levels of CDK1, MAPK11, and PDIA3 in patient blood. **(F)** Western blot analysis validated increased protein expression of CDK1, MAPK11, and PDIA3 in brucellosis. Original blots are presented in S2 Fig. **P < 0.01, ***P < 0.001.

Using 10-fold cross-validation, we evaluated the performance of the GBM and random forest models (S1 Fig). The GBM model achieved an average AUC of 0.960, while the random forest model achieved an AUC of 0.994 (Fig 5A). These results demonstrate strong diagnostic accuracy for distinguishing brucellosis from healthy controls. By applying GBM and Random Forest models, we evaluated the diagnostic value of module genes. Specifically, CDK1, MAPK11, and PDIA3 were identified as marker genes due to their high importance scores in the models (Fig 5B and C). In the GSE69597 dataset, the expression levels of CDK1, MAPK11, and PDIA3 were significantly higher in patients with brucellosis compared to the control group (Fig 5D).

RT-qPCR validation confirmed that the mRNA levels of CDK1, MAPK11, and PDIA3 were significantly elevated in brucellosis patients compared to the control group (Fig 5E). Similarly, Western blot experiments revealed that the protein expression levels of CDK1, MAPK11, and PDIA3 were significantly higher in patients with brucellosis than in the control group (Fig 5F, S2 Fig).

## Discussion

This study, through in-depth analysis of the GSE69597 dataset and validation with clinical samples, revealed significant gene expression differences and changes in the composition of immune cells between patients with brucellosis and healthy control groups, offering new insights into the cellular and molecular mechanisms of Brucella infection. The identification of 4924 DEGs reflects the extensive impact of Brucella infection on host cell gene expression. These DEGs reveal a potential complex regulatory network involved in the disease process, providing important clues for further exploration of the molecular mechanisms underlying brucellosis.

Analysis using CIBERSORT and flow cytometry indicated that the abundance of CD8 + T cells and NK cells is significantly higher in patients with brucellosis compared to healthy controls, while the abundance of B cells and CD4 + T cells is lower. This finding is consistent with other studies, which have shown a lower proportion of CD4 + T cells and a higher proportion of CD8 + T cells in acute and chronic phases [9]. Activated CD8 + cytotoxic T lymphocytes (CTLs), through Fas or perforin-mediated cytotoxicity and IFN-γ secretion, contribute to protective immunity against Brucella [10]. This may reflect an adjustment of the host immune system following Brucella infection, particularly an enhanced cytotoxic immune response in an attempt to clear the pathogen. NK cells also play a significant role in the pathogenesis of brucellosis through early production of IFNγ and their cytotoxicity, which is suppressed during the acute phase of the disease [11]. Studies have shown that Brucella promotes IL-2 production and activates NK cells to release TNF-α, IFN-γ, and granulocyte-macrophage colony-stimulating factor, thereby interfering with the host’s innate immune response [6].

Furthermore, B cells promote Tfh responses, enhancing susceptibility to *Brucella* [12]. The presence of B cells boosts the production of IL-10 by T cells, adversely affecting the control of *Brucella* load [13]. A major determinant of the ostensibly ineffective T cell response during *Brucella* infection stems from the suppression of CD4 + T cell responses by B cells [14,15]. The reduction in CD4 + T cells, a primary defense source, reaffirms the notion that protection against brucellosis relies on CD4 + T cells [16]. The decrease in B cells and CD4 + T cells might indicate *Brucella*’s strategy to optimize its survival environment by interfering with host immune regulatory mechanisms.

On the other hand, through WGCNA, DEGs were clustered into 11 gene modules, with the Brown module appearing to correlate positively with the normal state and CD8 + T cell immunity, whereas the Greenyellow module was closely associated with the infection state. CD8 + T cell-mediated responses contribute to the prevention of Brucella through the secretion of IFN-γ, although their primary function is to eliminate infected cells through Fas-FasL interactions and/or the secretion of perforin and granzymes [17].

Functional enrichment analysis suggested that genes in the Brown module were mainly involved in the cell cycle, virus infection, and immune system processes. Genes in the Greenyellow module were primarily associated with apoptosis, the PI3K-Akt signaling pathway, and the Wnt signaling pathway. The cell cycle and infection of Brucella are coordinated, favoring replication and survival in various niches [18]. Inhibition of apoptosis is considered a virulence trait shared by many intracellular pathogens to ensure productive replication, with targeting Fas as a strategy to avoid apoptosis also observed in *Brucella* [19]. *Brucella* infections prevent apoptosis induction by maintaining pro-survival pathways and PI3K-Akt signaling [20]. The PI3K-AKT-mTOR pathway is involved in regulating cellular events such as growth, adhesion, and proliferation, facilitating *Brucella* invasion and replication [7]. Positive regulation of Wnt/β-Catenin expression promotes bacterial replication and inhibits apoptosis [21]. The identification of these modules not only provides new perspectives for understanding the molecular mechanisms of brucellosis but also highlights potential therapeutic targets, especially those involved in regulating host immune responses and cell survival.

Importantly, through the analysis with GBM and Random Forest models, CDK1, MAPK11, and PDIA3 were further identified as potential diagnostic marker genes for brucellosis. They stood out not only in statistical models but were also validated by RT-qPCR and Western blot experiments, showing significantly increased expression in patients with brucellosis. CDK1, one of the key regulators of the G2/M checkpoint, is expressed in many cells and plays a crucial role in cell cycle control [22]. The upregulation of CDK1 mediates the activation of Wnt/β-Catenin to promote cell proliferation and counteract infection-induced cell death [23]. MAPK11, as one of the subunits of MAPK, its overexpression enhances the proliferation and colony formation ability of endometrial carcinoma cells [24]. PDIA3 has pleiotropic functions, proven to promote the proliferation and invasion of cancer cells, and can directly participate in the replication of the influenza virus [25]. Therefore, CDK1, MAPK11, and PDIA3 may not only have application value in the diagnosis of diseases but also suggest their key roles in disease progression.

Despite providing new insights into the molecular mechanisms of brucellosis, this study has some limitations. First, the analysis based on a single public dataset might be limited by data quality and sample size. Second, although we identified genes and immune cell subgroups changes associated with the disease state, these findings need to be validated in larger, independent cohorts. Furthermore, future research should explore how these differentially expressed genes and changes in immune cells specifically affect the development and progression of brucellosis and whether they can serve as potential targets for therapeutic intervention.

## Conclusion

Overall, this study provides a new perspective on understanding the cellular and molecular mechanisms of brucellosis by integrating gene expression profiling and immune cell scoring, laying the groundwork for developing novel diagnostic methods and therapeutic strategies. Future research should focus on the functional validation and clinical application of these findings to improve the management and treatment of brucellosis.

## Supporting information

S1 Fig The performance of the GBM and random forest models evaluated using 10-fold cross-validation. (TIF)

S2 Fig Images of protein expression of CDK1, MAPK11, and PDIA3 in brucellosis detected by Western blot. (TIF)

S1 Table Sequences of primers for qRT-PCR. (DOCX)
