# Supplementary figures and images for: Exploring the molecular mechanisms and immune cell responses in brucellosis: Insights from gene expression profiles and immune cell scores
Source: PLoS One. 2025 Sep 25;20(9):e0330840. doi: 10.1371/journal.pone.0330840 (PMC12463236; doi:10.1371/journal.pone.0330840)

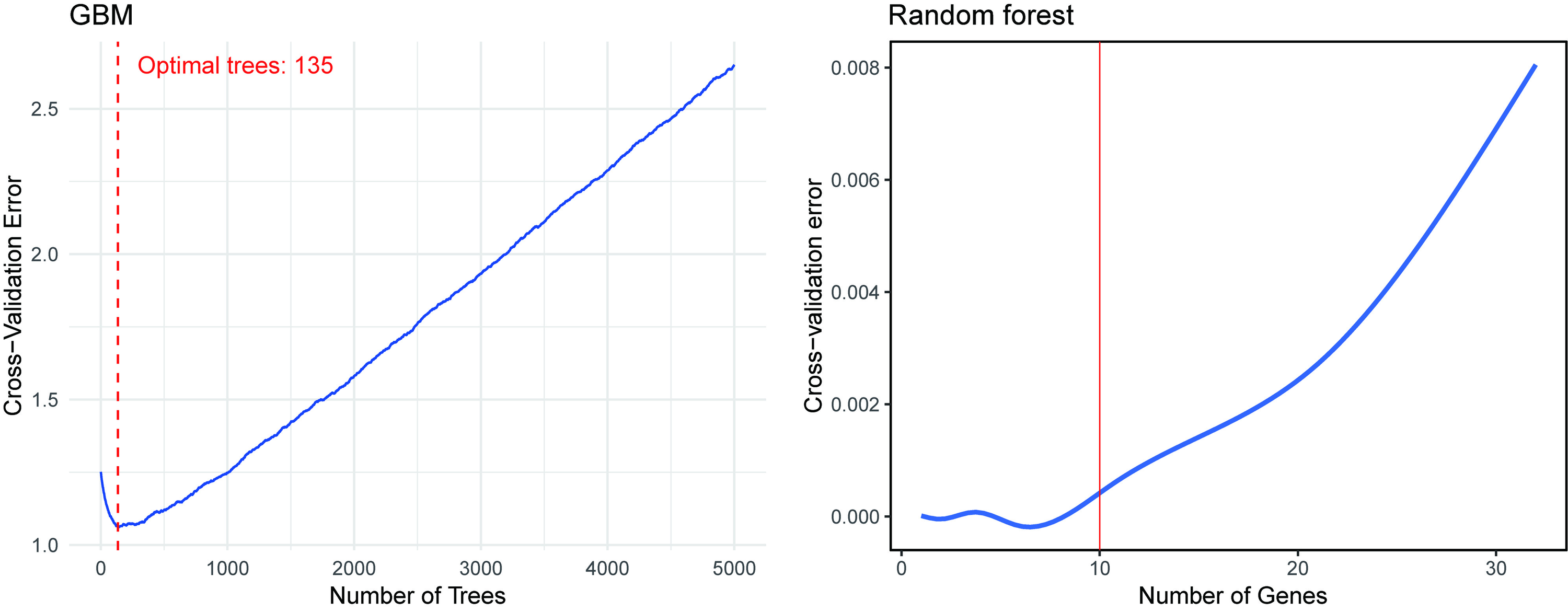

Supplement: S1 Fig — (TIF) [file pone.0330840.s001.tif]

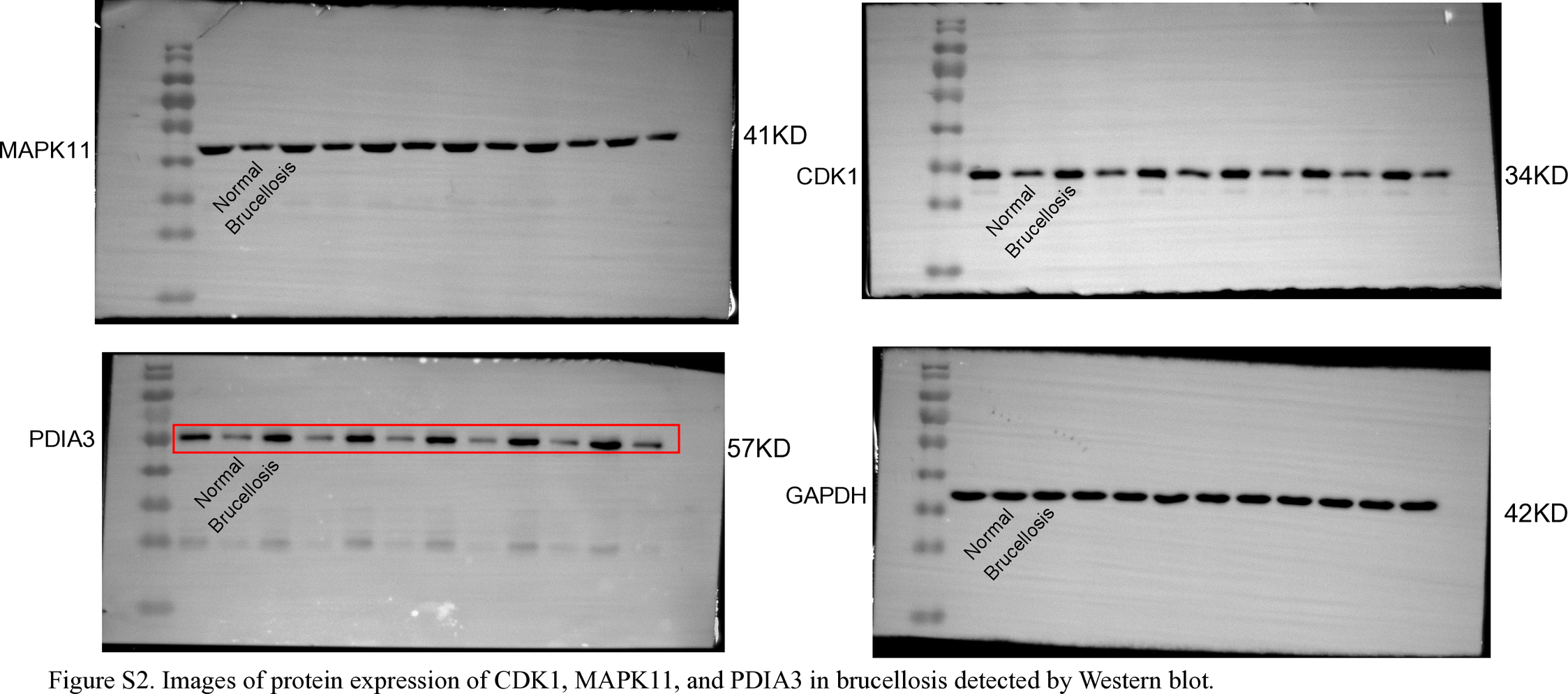

Supplement: S2 Fig — (TIF) [file pone.0330840.s002.tif]
