# Supplementary material for: Exploring the molecular mechanisms and immune cell responses in brucellosis: Insights from gene expression profiles and immune cell scores
Source: PLoS One. 2025 Sep 25;20(9):e0330840. doi: 10.1371/journal.pone.0330840 (PMC12463236; doi:10.1371/journal.pone.0330840)
Supplement: S1 Table — (DOCX) [file pone.0330840.s003.docx]

Table S1. Sequences of primers for RT-qPCR.

| Genes | Primers (5’-3’) |
| --- | --- |
| *H-MAPK11* | F: 5′-AGCACCTGAAGCACGAGAACG-3′ |
|  | R: 5′-CCTGGCACTTGACGATGTTGTTC-3′ |
| *H-PDIA3* | F: 5′-TGCTAGAACTCACGGACGACAAC-3′ |
|  | R: 5′-AGCTTCATACTCAGGTGCAAGTCTC-3′ |
| *H-CDK1* | F: 5′-CCAGGAAGCCTAGCATCCCATG-3′ |
|  | R: 5′-TGCCATTTTGCCAGAAATTCGTTTG-3′ |
| *GAPDH* | F: 5′-TGAAGGTCGGAGTCAACGGATTT-3′ |
|  | R: 5′-GCCATGGAATTTGCCATGGGTGG-3′ |
